# Creativity associated with the application of a motivational intervention programme for the teaching of dance at school and its effect on the both genders

**DOI:** 10.1371/journal.pone.0174393

**Published:** 2017-03-23

**Authors:** Diana Amado, Pedro Antonio Sánchez-Miguel, Pablo Molero

**Affiliations:** 1 Department of Didactics of Musical, Plastic and Corporal Expression. Faculty of Sport Sciences, University of Extremadura, Cáceres, Spain; 2 Department of Didactic of Musical, Plastic and Corporal Expression, Faculty of Teacher Training, University of Extremadura, Cáceres, Spain; Waseda University, JAPAN

## Abstract

The current study reviews processes of teaching-learning based on creativity, with the application by teachers of several strategies to support the need for autonomy, competence, and relatedness. The aim is to learn the effect of pupil’s gender on their motivational level and the psychological consequences that might arise in the cognitive, affective, and behavioural domains. A quasi-experimental study was carried out at four schools in Mexico, with 12 physical education teachers and 40 natural groups of pupils aged between 11 and 17 (*M* = 13.17). The groups were randomly assigned to either an experimental group (24 groups, 447 pupils) or a control group (16 groups, 474 pupils). A prior training programme was carried out with the teachers in the experimental group to enable them to support the psychological need for autonomy, competence, and relatedness. Initial and final measurements were taken in both groups, and the results revealed that independently of the programme used, girls showed higher motivation and positive psychological consequences in the teaching of dance compared to the male participants. In conclusion, it is important to continue with research and set a methodology that addresses those differences, dedicating the necessary time and treatment to resolve their questions and necessities.

## Introduction

Art and pedagogy are supported by the same educative principles, respect for the pupils’ questions and guiding them to find their own answers [1], and so, both disciplines must coexist in the teaching of dance. In this regard, the pedagogy that develops the activities of the artistic-expressive dimension has led teachers to suggest ideas without imposition, use active/inductive methods, respond differently to mistakes and negative experiences, promote discussion during feedback, maintain a receptive style, and have the attitude to learn at the same time as pupils; in sum, let teachers to experiment with a creative teaching-learning process [2], which is shown by current intervention programmes that are developing for dance teaching [3].

The activities of the artistic-expressive dimension stimulate motor creativity, using generally open premises that help to generate singular answers and encourage frequent improvisation by pupils [4]. Hence, improvisation is used in dance as a technique to develop divergent thought [5, 6]. These premises might be shown through different teaching procedures, such as an adequate level of practice and conditioning, the use of feedback, the stimuli of critical thought, the use of skills to perform, or giving different guidelines [7]. During dance classes, practice and conditioning might be easily changed through interaction with other pupils or object manipulation, and tasks can be shown through the use of metaphors or other verbal resources.

In accordance with this creative approach, which combines the pedagogy dimension with the artistic-expressive dimension, the following questions emerge: would the pupils’ expressive and creative capacities be improved by the teacher’s expressive and creative capacity? Can we develop these skills during teacher training? Dance and education domains share similar characteristics: a common interest with respect to motivating others and being more effective in the learning-teaching process. Hence, as described in detail previously [8], creativity work has been associated with motivation [9,10], emphasising in several studies the teacher’s role as a key element in the type of motivation shown by pupils in physical education [11–14].

These works are focused on the study of motivation through the postulates of Self-Determination Theory [15,16], which considers that motivation is a continuum that ranges from higher to lower levels of self-determination, which refers to the degree to which behaviors are volitional and involuntary. The higher degree of self-determination is intrinsic motivation, defined by performing an activity that produces a pleasant feeling when participating in the activity. This type of motivation has been associated with positive consequences such as enjoyment, effort, usefulness, or adherence to physical education [17–19], and also with the development of positive behaviour in physical education [20]. The second degree of motivation is extrinsic motivation, in which the behaviour for an activity is unrelated to the person themselves and appears to be related to outside interests. In this type of motivation there are different sublevels, listed in descending order with reference to the self-determination level: identified regulation, introjected regulation, and external regulation [16]. Finally, the lowest level of self-determination is amotivation, in which the subject does not know exactly why to keep practicing.

In accordance with this theory, the type of motivation shown by a person depends on the satisfaction of their basic psychological needs [15], on autonomy (the feelings of volition and the sense that the individual has personal control of his or her own behaviour), competence (the development of an effective interaction and sense of accomplishment with the environment), and relatedness (the feeling of connection and the development of belongingness within the social context in which the activity is performed). Therefore, it might be possible increase pupils’ motivation and their positive consequences in physical education if the teacher uses a more creative methodology that includes strategies to support the satisfaction of these three needs [21–23]. Specifically, a teacher can use specific autonomy support strategies, refer to the use of cognitive teaching styles to give the students the responsibility of choosing the tasks that they will perform as well as allow pupil freedom in decision-making [24]. Likewise, competence support strategies are aimed at optimising pupils’ perception of their skill through activities that are adjusted to their level and that provide a sufficient time frame to achieve the proposed aims, while providing positive feedback and acknowledging their efforts and progress [25–26]. Finally, relatedness support refers to the teacher’s resources for creating learning environments that promote feelings of inclusion, integration, trust, and respect among the classmates [26].

Nevertheless, when testing pupil motivation, it is crucial to study the effect of gender, because whereas boys show a more self-determined motivation towards physical education in general [27, 21], girls show less enjoyment, interest, and pleasure during these classes [28,29]. Conversely, regarding dance and artistic-expressive activities, girls show a greater interest than boys in this type of activity [30, 31]. The teaching of dance is very particular and the current tendency is to set programmes where pupil creativity is promoted, because it is shown that if an adequate structure and set of classes is performed, we are helping to establish social bonds between the genders where they have to help each other, avoiding comparison between them [3].

Therefore, the aim of this study is to learn whether gender differences exist in the motivational processes (the perception of teacher support of the basic psychological needs, the perception of the satisfaction of these needs, and the self-determination level), in the cognitive (usefulness of dance), affective (enjoyment and effort), and behavioural (positive behaviour) consequences that pupils show, after applying a dance teaching programme in the school developed by the teacher that is based on creativity and the development of strategies to support autonomy, competence, and relatedness.

## Method

### Ethics statement

Insofar as ethical rules are concerned, the study previously received the approval of the Ethics Committee of the University of Extremadura. All participants were treated in agreement with the ethical guidelines of the American Psychological Association with respect to consent, confidentiality, and anonymity of the answers. Before carrying out the research study, all involved were informed about the process that they were going to follow, placing emphasis on the fact that participation was voluntary and that the data would be dealt with in a confidential manner. Moreover, an informed written consent was obtained from the parents and the headteachers of the schools on the behalf of the minors/child participants involved in the study.

### Participants

Twelve physical education teachers of the “Learning with Dance” programme in Mexico participated in the study. The study was organised by ConArte (International Consortium of School and Art) and included the twelve teachers’ 921 (female *n* = 421 and male *n* = 500) pupils, aged between 11 and 17 (*M* = 13.17 years, *SD* = 1.12), from four schools in Mexico City. All the pupils were in the 1^st^, 2^nd^, and 3^rd^ grade and were selected according to the classes they were grouped into (A, B, C, D or E). The groups were divided into 24 experimental groups with a total of 447 pupils and into 16 control groups with a total of 475 pupils.

All the participants belonged to the “Learning with Dance” program, developed by “ConArte” (International Consortium of School and Art). Within this organisation, the “Learning with Dance” programme provides an interdisciplinary dialogue between dance and music from a methodology designed specifically for the public classroom and/or community spaces. Its purpose is to provide an education in art in the cognitive, social-affective, and psychomotor domains, with the aim of promoting the artistic experience. To achieve this purpose, it requires the training of teachers in the arts of dance and music with a methodology focused on the specific contexts of teaching practice for schools and vulnerable people.

### Instruments

#### Perception of basic psychological needs support

An adaptation of the dance and corporal expression context of the Questionnaire of Support to the Basic Psychological Needs [32] was used with the pupils. This instrument has 12 items preceded by the heading: “In the dance lessons I have notice that our teacher…”, and grouped into three factors (four items per factor). These factors are autonomy support (e.g.: “Asks us frequently about our preferences”), competence support (e.g.: “Encourages us to believe in our ability to perform the activities well”),and relatedness support (e.g.: “Is always improving the relationship the pupils have with each other”).

#### Perception of basic psychological needs satisfaction

To measure the pupils’ satisfaction of their basic psychological needs, a version of the Basic Psychological Needs Measurement Scale [33] translated into Spanish [34] was used. It was adapted by modifying the wording of the initial sentence, transferring it to the context of dance education. The instrument was preceded by the heading: “In the dance lessons that we have received…”, followed by 12 items (four per factor), which measure the satisfaction of autonomy (e.g.: “The way the exercises are carried out coincide perfectly with the way in which I want to do them”), competence (e.g.: “I feel that I have progressed greatly with respect to the final objective that I had set out for myself”), and relatedness (e.g.: “I feel very comfortable when I carry out the exercises with the other companions”).

#### Level of self-determination

To evaluate the type of motivation of the pupils, the Questionnaire on Motivation in Dance and Corporal Expression [35] was used. This tool is headed by the statement: “I have participated in the dance lessons that we have received…” followed by 20 items grouped into five factors (four per factor) that measure intrinsic motivation (e.g.: “Because they are fun”), identified regulation (e.g.: “Because I can learn skills that I could use in other areas of my life”), introjected regulation (e.g.: “Because it is what I must do to feel good”), external regulation (e.g.: “Because it is well looked upon by the teacher and companions”), and amotivation (e.g.: “But, I do not understand why we have to have this content in Physical Education”).

#### Usefulness

To measure the pupils’ perception of usefulness of dance in education, the adaptation of an instrument created by [36] was used. This is comprised four items that refer to the pupils’ perception of the benefits of sporting practice and to its importance in their lifestyle. The adaptation consisted of modifying the wording of certain terms of the items, transferring them to the context of dance education. The items used were the following: “In general, to what extent do you believe dance is useful for you?”; “For you, to what extent is it important to be good at dance?”; “Compared with the other content delivered at the school, to what extent do you find the dance useful for you?”; “Compared with the majority of your other activities, to what extent is it important for you to be good at dance?”.

#### Enjoyment and effort

To evaluate the enjoyment and effort expressed by pupils within the context of dance education, a version of the Intrinsic Motivation Inventory [37] translated into Spanish and adapted to physical education by [38] was used. This instrument was adapted by modifying the wording of certain terms of the items, transferring them to the context of dance education. This instrument comprised four factors: enjoyment-interest, perception of competence, effort-importance, and stress-pressure, but to carry out this study, only two of these factors were selected: enjoyment-interest and effort-importance. The instrument was headed by the statement: “In the dance lessons that we have received…,” followed by nine items divided into two factors that measure enjoyment-interest (five items, e.g.: “I have a lot of enjoyment dancing”) and effort-importance (four items, e.g.: “I have made every effort dancing”).

#### Positive behaviors

To measure the pupils’ positive behaviors within the context of dance education, an adaptation of the Positive Behaviors in Physical Education Questionnaire [39] was used. The adaptation was to modify the wording of the initial sentence, transferring it to the context of dance education. The instrument was preceded by the heading: “In the dance lessons that we have received…”, followed by 18 items presented dichotomously. These measured respect for the facilities (e.g.: “I do not respect the facilities of the center” and “I respect the facilities of the center”), effort evaluation (e.g.: “It is not important to work hard in order to improve” and “It is the most important to work hard in order to improve”), tolerance (e.g.: “I find it difficult to accept my peers with a lower level of ability than me” and “I accept my peers regardless of their level of ability”), cooperation (e.g.: “I do not like to participate in group activities” and “I love to participate in group activities”), and self-control (e.g.: “When I lose my patience I get aggressive” and “When I lose my patience I know how to control myself”).

The answers to all of these instruments were carried out on a 5-point Likert-type scale, where 1 corresponded to “strongly agree” with the negative statement and 5 corresponded to “strongly agree” with the positive statement, except in the case of usefulness. This instrument was answered on a 5-point Likert type scale, where 1 corresponded to “Nothing” and 5 to “A lot.”

### Procedure

Once the ethics norms of the study were set, the investigation began with the development of a training programme in dance education. The selected contents were:

Theme 1. Dance and corporal expression at the school:

1.1 The concept of educational dance
1.2 The style technique
1.3 The creative technique
1.4 Movement factors
1.5 Ways of corporal expression
1.6 The literacy process of the body language

Theme 2. Body

Theme 3. Weight

Theme 4. Contact

Theme 5. Space

Theme 6. Time

Theme 7. Intensity

Theme 8. Interaction

Theme 9. Methods for learning to create choreographies

Theme 10. The construction of the observatory’s sight

Theme 11. Didactics of the physical-artistic activities

11.1 Selection of proposals
11.2 The group dynamic, the constructivist reflection and the evaluation

The programme lasted 35 hours and it was developed with 21 teachers from the “Learning with Dance” programme, organised into groups of three people (one music teacher and two dance teachers). The programme began by distributing a document to each attendee in which the course content appeared distributed in a didactic unit of 12 sessions. Each session of the programme was based on the theoretical explanation given by the principal investigator and its subsequent practical application (see Table 1). Each practical part of each session was carried out by one of the groups formed of three people. These groups developed a practice class for the assistants, who had been previously trained and supervised by the principal investigator.

**Table 1:** Organisation of the programmes.

**WEEK 1**					
**Time**	**Monday**	**Tuesday**	**Wednesday**	**Thursday**	**Friday**
09:00–11:00	Content 1	Content 3	Content 5	Content 7	Review of content
11:00–11:30	Break	Break	Break	Break	Break
11:30–14:30	Content 2	Content 4	Content 6	Content 8	Review of content
**WEEK 2**					
**Time**	**Monday**	**Tuesday**	**Wednesday**	**Thursday**	**Friday**
09:00–11:00	Content 9	Content 11	Training ofStrategies	Training ofStrategies	Training ofStrategies
11:00–11:30	Break	Break	Break	Break	Break
11:30–14:30	Content 10	Review of content	Training of Strategies	Training of Strategies	Training of Strategies
16:30–20:30			Training of observers	Training of observers	Training of observers

Then, after receiving the training programme content, 12 teachers were selected, based on their level of ability and their availability, to teach the didactic unit the following year. Six of these 12 teachers were assigned to the experimental group, developing with them an intervention programme based on creativity and incorporating skills into their teaching style to increase pupil motivation. The other six teachers were assigned to the control group and only had to apply the content without including the programme.

The teachers in the experimental group had to apply a creative methodology as described in detail previously [8], where the pupils are the ones that start and assume responsibility for their learning whilst the teacher acts as a mentor in the teaching process (Kassing & Jay, 2003). It is based on the creativity or ability to create different motor and expressive responses when a problem arises (Kassing & Jay, 2003). Furthermore, they had to attend a seminar on the theory of self-determination, which stressed the importance of supporting the need for competence, autonomy, and relatedness. Subsequently, they were given a document in which every one of the strategies that must be applied in each of the 12 sessions of the didactic unit appeared. Several sessions were used to provide examples of how these strategies may be pedagogically applied (see Table 1). The strategies were taken from [40], in which each strategy appears that support the autonomy, competence, and relatedness needs, adapted to the dance context. These are as follows:

To support autonomy: use the democratic leadership style to foster pupils’ active participation; try not to control or put too much pressure on the pupils in order to reduce competitiveness; make it possible for them to choose some activities by specifying the rules; explain the objective of the activity so that there is greater engagement; use rewards related to practice and place emphasis on the pupils’ social skills.

To support competence: adapt the exercises to the pupils’ level; design activities where success is evaluated through intrapersonal instead of interpersonal indicators in order to foster effort and personal improvement; provide positive feedback so that pupils feel more secure and more aware of the triumphs they achieve; provide objectives and feedback regarding the process and not the result in order to increase the skill level perceived by the pupils; propose different and accessible short-, medium-, and long-term objectives that adapt to the pupils’ needs.

To support relatedness: set aside some moments during the session for pupils to be able to establish personal relations and deal with unrelated topics; form mixed gender groups to boost the integration of all pupils; educate the pupils in social skills so that they learn to show empathy towards each other and towards the teacher; create cooperative exercises in order to encourage pupils to make mutual and dynamic group decisions that provoke the exchange of opinions and establish strong and long-lasting relationships.

To ensure proper implementation of the intervention programme, a training programme with seven observers was developed (see Table 1). These observers were dance degree students who were interested in participating to acquire further knowledge of dance education. This training programme was similar to that which the experimental groups received in that it insisted on the correct/ incorrect application of each strategy.

Once the various training programmes had been developed, we proceeded to the implementation. A quasi-experimental design was used, with 40 natural groups already established by a school centre, so it was not possible to respect randomisation. These groups were divided into 16 control groups (*n* = 474) and 24 experimental groups (*n* = 447). Both groups received the same dance teaching programme, but with the experimental group a motivational intervention programme based on creativity was also developed. The research was performed throughout the first term at a secondary education centre, with two weekly 50-minute sessions distributed throughout the week that took place in the gymnasium of the centre.

To collect the research data, two measurements were carried out, pre and post. The pre-measurement was carried out during the first session of each group, before this session was given, and the post-measurement was taken at the end of the process in both groups. Data collection consisted of administering a questionnaire to the pupils, which they completed in the classroom without the presence of the teacher and in a climate that enabled them to concentrate without any type of distraction for 20 minutes. The principal investigator was present at all times to explain any doubts and make sure that the process was strictly followed.

### Data analysis

Data was analysed using the SPSS 18.0 statistical package. Different tests were conducted to determine the nature of the data, the K-S test for independent samples to verify the normality of the groups, the Rachas test for randomness, and Levene’s test for the homoscedasticity or equality between variances, and the nature of the data was parametric.

Firstly, an analysis of descriptive statistics and reliability was conducted with all of the study variables. Afterwards, a multivariate analysis of variance (MANOVA) with the data collected in the pre-test was developed, including the group (the control group and the experimental group) and gender (male, female) as independent variables, to observe the homogeneity of the sample. Finally, to observe the gender differences, a three-factor repeated measures analysis of variance was performed, with two inter-subject factors (two-level gender and two-level group) and one intra-subject factor (two-level time of measurement), with the aim of comparing the interaction between teaching technique and pupils’ gender in the pre-test and post-test.

## Results

### Descriptive statistics and reliability analysis

Table 2 shows the descriptive statistics of the study variables with the pre-test and post-test data. The scores obtained in the pre-test and the post-test were similar, with lower values in the post-test in all study variables. The average score in all cases was high, close to the maximum given in Likert scale. Kurtosis and skewness scores were acceptable, with all the values lower than 2, and also, in the reliability analysis, all the factors attained a Cronbach alpha level over .70.

**Table 2:** Descriptive statistics and reliability analysis of the study variables.

	PRE					POST				
VARIABLES	*M*	*SD*	*α*	*Skewness*	*Kurtosis*	*M*	*SD*	*α*	*Skewness*	*Kurtosis*
Competence support	3.83	.88	.75	-.75	.16	3.64	.91	.76	-.47	-.16
Autonomy support	3.65	.89	.72	-.48	-.35	3.48	.96	.75	-.41	-.37
Relatednes support	3.82	.87	.75	-.63	-.11	3.59	.93	.79	-.44	-.29
Competence satisfaction	3.79	.84	.71	-.69	.26	3.50	.91	.74	-.48	-.09
Autonomy satisfaction	3.52	.96	.77	-.52	-.32	3.33	.99	.77	-.31	-.48
Relatedness satisfaction	3.82	.92	.76	-.69	-.12	3.48	.98	.77	-.36	-.48
Intrinsic motivation	3.90	.93	.80	-.89	.30	3.60	.99	.80	-.58	-.20
Identified regulation	3.87	.88	.76	-.73	-.03	3.51	.99	.82	-.49	-.24
Introjected regulation	3.36	.87	.58	-.22	-.52	3.19	.90	.65	-.22	-.31
External regulation	3.62	.87	.64	-.42	-.34	3.33	.92	.66	-.24	-.38
Amotivation	2.59	.99	.64	.28	-.59	2.75	.99	.68	.13	-.57
Usefulness	3.65	.98	.84	-.74	-.15	3.41	1.06	.86	-.42	-.56
Enjoyment	3.61	.86	.66	-.60	-.01	3.51	.93	.75	-.41	-.31
Effort	3.61	.85	.63	-.63	.30	3.51	.90	.70	-.48	.00
Respect of facilities	4.00	.96	.76	-.95	.43	3.75	.96	.73	-.47	-.33
Effort Evaluation	3.99	.98	.71	-.95	.43	3.72	1.00	.68	-.49	-.37
Tolerance	3.80	.94	.71	-.75	.30	3.61	.89	.65	-.29	-.28
Cooperation	3.80	1.04	.69	-.74	-.06	3.60	1.01	.66	-.40	-.42
Self-control	3.68	.97	.71	-.67	.10	3.63	.91	.67	-.42	-.07

### Preliminary analysis

We used a MANOVA to examine the homogeneity of groups in the pre-test, including the group (the control group and the experimental group) and the gender (male, female) as independent variables, and the perception of basic psychological needs support, the perception of basic psychological needs satisfaction, the level of self-determination, usefulness, enjoyment, effort, and positive behaviour of the pupils towards dance as dependent variables.

To begin with, regarding the pupils’ perception of basic psychological needs support, significant differences were found at the multivariate level in agreement with the gender (*F* (1, 919) = 6.92, *p* = .00; *n*^*2*^ = .02), whilst no significant differences were obtained with respect to the group (*F* (1, 919) = .60, *p* = .61; *n*^*2*^ = .00) and with respect to the group-gender interaction (*F* (3, 917) = 1.46, *p* = .22; *n*^*2*^ = .00). The subsequent analysis of the inter-subject effects provoked by gender revealed significant differences in autonomy support (*p* < .01), competence support (*p* < .01), and relatedness support (*p* < .01), where the female gender recorded higher scores than the male gender.

With respect to basic psychological needs, significant differences were found at the multivariate level in agreement with the gender (*F* (1, 919) = 7.60, *p* = .00; *n*^*2*^ = .02) and the group (*F* (1, 919) = 3.25, *p* = .02; *n*^*2*^ = .01), whilst no significant differences were obtained with respect to the group-gender interaction (*F* (3, 917) = 1.12, *p* = .34; *n*^*2*^ = .00).The subsequent analysis of the inter-subject effects provoked by gender revealed significant differences in autonomy satisfaction (*p* < .01), competence satisfaction (*p* < .01), and relatedness satisfaction (*p* < .01), where the female gender recorded higher scores than the male gender. Moreover, the subsequent analysis of the inter-subject effects provoked by the group revealed significant differences in relatedness (*p* = .01), where the control group recorded higher scores than the experimental group.

Following this, with respect to the type of motivation, the data analysis indicated statistically significant differences at the multivariate level, in agreement with gender (*F* (1, 919) = 11.60, *p* = .00; *n*^*2*^ = .06), whilst no significant differences were obtained with respect to the group (*F* (1, 919) = .76, *p* = .58; *n*^*2*^ = .00) or with respect to the group-gender interaction (*F* (3, 917) = 1.40, *p* = .22; *n*^*2*^ = .01). The subsequent analysis of the inter-subject effects provoked by gender revealed significant differences in intrinsic motivation (*p* = .05), identified regulation (*p* < .05), introjected regulation (*p* = .01), external regulation (*p* = .00), and amotivation (*p* < .05), where the female gender recorded higher scores than the male gender in all variables except amotivation.

Finally, focusing on the different consequences such as usefulness, enjoyment/effort, and positive behaviour of the pupils towards dance significant differences were found at the multivariate level depending on the gender (*F* (1, 919) = 10.72, *p* = .00; *n*^*2*^ = .06). These differences did not appear with respect to the group (*F* (1, 919) = .69; *p* = .63; *n*^*2*^ = .00) or with respect to the group-gender interaction (*F* (3, 917) = 1.21; *p* = .30; *n*^*2*^ = .01). The subsequent analysis of the inter-subject effects caused by gender reveal significant differences in usefulness (*p* = .05), enjoyment (*p* < .05), effort (*p* < .05), and positive behaviour (*p* = .01), where the female gender recorded higher scores in all cases with respect to the male gender.

Therefore, after performing the preliminary analysis it can be stated that the groups were heterogeneous prior to the intervention.

### Analysis of the gender differences

We then examined the existing differences between the dependent variables included in the study between four gender/group series. To this end, a three-factor repeated measures analysis of variance was performed, with two inter-subject factors (group and gender) and one intra-subject factor (time of measurement) (Table 3). This analysis showed significant principal effects for gender at the inter-subject level (*F* (1, 919) = 6.46, *p* = .00; *n*^*2*^ = .08) but no significant effects were found for the group (*F* (1, 919) = .90, *p* = .55; *n*^*2*^ = .01) and for group x gender interaction (*F* (3, 917) = 1.29, *p* = .22; *n*^*2*^ = .02). The subsequent analysis of the inter-subject effects caused by gender reveal significant differences in the perception of basic psychological needs support (*p* < .01), the perception of basic psychological needs satisfaction (*p* < .01), intrinsic motivation (*p* < .01), identified regulation (*p* < .01), amotivation (*p* < .01), usefulness (*p* < .01), enjoyment (*p* < .01), effort (*p* < .01), and positive behaviour (*p* < .01), where the female gender recorded higher scores in all cases with respect to the male gender except in amotivation, where the male gender recorded higher scores than the female gender.

**Table 3:** Gender differences in repeated measures analysis of variance and descriptive statistics for the dependent variables.

	Male gender				Female gender						
	Control group		Experimental group		Control group		Experimental group				
	Pre-test	Post-test	Pre-test	Post-test	Pre-test	Post-test	Pre-test	Post-test			
	*M±SD*	*M±SD*	*M±SD*	*M±SD*	*M±SD*	*M±SD*	*M±SD*	*M±SD*	*F*	*p*	*η*^*2*^
BPN Support	3.72±.82	3.52±.88	3.60±.80	3.50±.82	3.91±.78	3.62±.92	3.89±.79	3.67±.86	.04	.00	.00
BPN Satisfaction	3.66±.85	3.39±.86	3.55±.78	3.45±.82	3.86±.75	3.36±.92	3.83±.78	3.54±.87	.21	.00	.00
Intrinsic motivation	3.76±.96	3.53±1.03	3.67±.97	3.53±.95	4.12±.86	3.56±1.05	4.12±.85	3.79±.93	.73	.00	.00
Identified regulation	3.76±.88	3.43±1.03	3.68±.89	3.48±.93	4.07±.85	3.50±1.04	4.01±.86	3.63±.95	.19	.00	.00
Introjected regulation	3.32±.90	3.20±.90	3.23±.82	3.22±.85	3.41±.87	3.12±.95	3.51±.86	3.20±.91	.90	.15	.00
External regulation	3.67±.89	3.37±.97	3.50±.88	3.38±.89	3.63±.89	3.21±.96	3.70±.84	3.34±.87	.66	.82	.00
Amotivation	2.65±.98	2.83±1.05	2.75±.96	2.90±.96	2.40±.97	2.64±1.00	2.45±1.00	2.58±.94	.23	.00	.00
Usefulness	3.49±1.06	3.26±1.10	3.42±.97	3.32±1.01	3.88±.92	3.52±1.12	3.93±.84	3.57±1.00	.68	.00	.00
Enjoyment	3.52±.88	3.50±.94	3.44±.90	3.43±.92	3.74±.78	3.52±.98	3.81±.80	3.59±.90	.01	.00	.00
Effort	3.52±.91	3.46±.97	3.50±.91	3.51±.84	3.73±.75	3.51±.93	3.73±.78	3.56±.86	.03	.00	.00
Positive behaviour	3.87±.82	3.61±.80	3.69±.78	3.58±.75	3.96±.86	3.80±.84	3.95±.81	3.70±.81	3.02	.00	.00

Note: BPN: Basic Psychological Needs.

At the intra-subject level, no significant effects were found for time of measurement x group interaction (*F* (3, 917) = 1.25, *p* = .24; *n*^*2*^ = .02), for time of measurement x gender interaction (*F* (3, 917) = 1.63, *p* = .08; *n*^*2*^ = .02), and for time of measurement x group x gender interaction (*F* (7, 913) = .74, *p* = .71; *n*^*2*^ = .01).

## Discussion

The aim of this research was to assess whether gender differences exist in the motivational processes (the perception of teacher support to the basic psychological needs, the perception of satisfaction of these needs, and the self-determination level), in cognitive (the usefulness of dance), affective (enjoyment and effort), and behavioural (positive behaviors) consequences that pupils show, after apply a dance teaching programme in school developed by the teacher, based on creativity and the development of strategies to support autonomy, competence, and relatedness.

After the research was conducted, statistical analysis showed the existence of significant differences regarding gender, but these differences were not associated with the programme they belonged to (the control or experimental group), not with the time of measurement (pre-test or post-test). Therefore, results demonstrated that, independently of the application of an intervention programme based on the creativity and orientated to promote pupils’ motivation, girls showed a greater perception of teacher support of their basic psychological needs, higher satisfaction of those needs, greater self-determined motivation (intrinsic motivation and identified regulation), a higher perception of the usefulness of dance, greater enjoyment, greater effort, and more positive behaviour than boys regarding educative dance. Nevertheless, boys showed greater amotivation than girls towards this discipline.

In line with these results, other studies have revealed the existence of differences between the genders in the motivation shown towards dance, and so, girls always show greater motivation and interest in this discipline [30,31], whereas boys revealed less motivation because they consider that it is an eminently feminine discipline, and they have valued themselves with fewer qualities than the female gender to develop this activity for social and cultural reasons [41,42]. Therefore, the outcomes found were quite relevant because they let us explain that differences emerged in motivation and interest between boys and girls respecting dance, there was a strong tendency of a feeling and an unshakable perception over time, and this is possibly related to the ideas that have traditionally existed about this discipline. According to this issue, research has demonstrated that boys and girls usually show competence and motivation in those physical activities that they consider adequate regarding their gender [43] and this aspect will determine their choice of physical activities and their answers to certain activities that are taught in physical education [44].

As described in detail previously [8], boys and girls are aware of their social expectations in terms of their participation in physical and sport activities, while boys traditionally show a desire for physical contact sports as central experiences when establishing an acceptable masculine identity[45],girls have a greater preference for artistic-expressive activities such as dance [30,31]. Accordingly, contrary to what happens in the teaching of dance, in the domain of physical education in general, investigations have suggested that girls had lower motivation [28, 29, 21] and showed less enjoyment and commitment during these classes [46,47], possibly because this subject has traditionally focused the learning on competence and physical attitude [48], and the methodology of learning used in the curriculum has followed a more traditional perspective focused on the teacher, which has led to physical education being defined as hegemonic, orientated to the sport and the competitive masculine culture, and being underestimated in its social, cognitive, and affective aspects [29, 49,47].

As can be seen, the main limitation found in this study consisted of the complexity of changing this tendency, and maybe due to this aspect, a motivational intervention programme is not enough to decrease the differences in the interest of both genders regarding dance. This assessment revolves around the stereotypes created around dance, and even more in the Mexican context that this research was developed, characterised by behaviour typical of a socially unshakable patriarchal and sexist education system [50].

Possibly, with the aim of achieving success in the desired effects of intervention, a methodology might be set that attends to these differences and gives time and the necessary approach to resolve their questions and necessities. And certainly, this methodology is based on creativity [8] because it is the way that teachers try to better approach the pupils’ preferences and connect with their aims, content, and procedures [8, 51, 52]

But creativity in the domain of dance requires a large amount of pupil comprehension because it is a principle that might decrease their competence and motivation [53] due to the difficulty of this type of content, which resides in the performances that teachers have to develop, such as pupil autonomy in front of their peers and using uncommon motor patterns [54]. Hence, one of the conclusions that we can take from this study is that, although this research has used a creative methodology to develop the content and increase the motivation of both genders, this purpose was not achieved, because it requires continuity and the time dedicated to this content in the school was limited to only a one hour class of dance per week. The increase of the frequency, as well as the length and application of this content in different educative levels, might promote a significant improvement in the motivation and assessment of the activity on the part of the male participants, but further investigation is necessary regarding this issue.

Moreover, another limitation that was found was the length of the teacher training in the motivational strategies: it was insufficient according their own statements, because sometimes they felt unsteady in the application of the strategies due to the relationship of some dance content modified by the pupils’ background and the conflicts that might appear in the classroom. Thereby, it might be interesting in the future to extend such training to promote the comprehension and the adaptation to the learning situations regarding conflicts.

Therefore, another conclusion is the importance of promoting the initial training of teachers with regard to the methodological treatment of dance content in school, with the aim of improving the teaching-learning process and regularising this type of content within any General Educative System, independent of the society in which it is applied.
