# Correction: Creativity associated with the application of a motivational intervention programme for the teaching of dance at school and its effect on the both genders

**DOI:** 10.1371/journal.pone.0178891

**Published:** 2017-05-30

**Authors:** Diana Amado, Pedro Antonio Sánchez-Miguel, Pablo Molero

There are errors in the Funding section. The correct funding information is as follows: This study was carried out thanks to the contribution of the Ministry of Economy and Infrastructure of the Board of Extremadura through the European Regional Development Fund. The funders had no role in study design, data collection and analysis, decision to publish, or preparation of the manuscript.

## References

[1] Amado D, Sánchez-Miguel PA, Molero P (2017) Creativity associated with the application of a motivational intervention programme for the teaching of dance at school and its effect on the both genders. PLoS ONE 12(3): e0174393 https://doi.org/10.1371/journal.pone.0174393 28333990 10.1371/journal.pone.0174393 PMC5363940

